# Trends, determinants, and newborn mortality related to thermal care and umbilical cord care practices in South Asia

**DOI:** 10.1186/s12887-019-1616-2

**Published:** 2019-07-22

**Authors:** Lindsay Mallick, Jennifer Yourkavitch, Courtney Allen

**Affiliations:** 1 The Demographic and Health Surveys (DHS) Program, Avenir Health, 530 Gaither Road, Suite 500, Rockville, MD 20850 USA; 2 The DHS Program, ICF, 530 Gaither Road, Suite 500, Rockville, MD 20850 USA

**Keywords:** Newborn care, Thermal care, Hygienic cord care, Newborn mortality

## Abstract

**Background:**

Although child mortality has decreased over the last several decades, neonatal mortality has declined less substantially. In South Asia, neonatal deaths account for the majority of all under-five deaths, calling for further study on newborn care practices. We assessed five key practices: immediate drying and wrapping, delayed bathing, immediate skin-to-skin contact after birth, cutting the umbilical cord with a clean instrument, and substances placed on the cord.

**Methods:**

Using data from Demographic and Health Surveys conducted in Bangladesh, India, and Nepal between 2005 and 2016, we examined trends in coverage of key practices and used multivariable logistic regression to analyze predictors of thermal care and hygienic cord care practices and their associations with neonatal mortality among home births. The analysis excluded deaths on the first day of life to ensure that the exposure to newborn care practices would have preceded the outcome. Given limited neonatal mortality events in Bangladesh and Nepal, we pooled data from these countries.

**Results:**

We found that antenatal care and skilled birth attendance was associated with an increase in the odds of infants' receipt of the recommended practices among home births. Hygienic cord care was significantly associated with newborn survival. After controlling for other known predictors of newborn mortality in Bangladesh and Nepal, antiseptic cord care was associated with an 80% reduction in the odds of dying compared with dry cord care. As expected, skilled care during pregnancy and birth was also associated with newborn survival. Missing responses regarding care practices were common for newborns that died, suggesting that recall or report of details surrounding the traumatic event of a loss of a child may be incomplete.

**Conclusions:**

This study highlights the importance of maternal and newborn care and services for newborn survival in South Asia, particularly antenatal care, skilled birth attendance, and antiseptic cord care.

**Electronic supplementary material:**

The online version of this article (10.1186/s12887-019-1616-2) contains supplementary material, which is available to authorized users.

## Background

In 2016, an estimated 2.6 million children died within the first 28 days after birth, an average of about 7,000 every day [1]. Newborn (neonatal) mortality—deaths within the first 28 days—currently accounts for 59% of all under-five mortality in South Asia [1]. While mortality at age 1–59 months declined globally by 62% between 1990 and 2016, the neonatal mortality rate (NMR) declined by only 49% [1]. The main causes of newborn death are premature birth (and consequently, low birthweight), intrapartum-related birth complications, and infections [2]. Life-saving interventions during the intrapartum period include antibiotics for premature rupture of membranes, corticosteroids for preterm labor, detecting and managing breech births and multiple pregnancy (twins, triplets, etc.), monitoring labor to identify complications, and clean birth practices [3, 4]. Recommended newborn care practices that reduce mortality include newborn resuscitation, immediate and exclusive breastfeeding, preventing and managing hypothermia, kangaroo mother care for low birthweight infants, and community case management of pneumonia [3].

Skilled care at birth and delivery in a facility can help avert preventable complications [3, 5]. Although the use of health facilities and a skilled birth attendant (SBA) for deliveries has increased, these interventions have contributed to only minimal reductions in maternal and neonatal deaths. The low impact of SBAs may be attributable to selection bias: women with higher-risk pregnancies seeking out facility delivery where most births are attended by SBAs [6, 7]. These newborns have lower odds of surviving, offsetting the hoped-for reduction in mortality [8]. Even though the benefit of delivering in a health facility is well-known, and facility birth is encouraged through national policies, women continue to deliver at home. These women are impeded by lack of access to facilities in terms of distance or cost, or fear of poor quality of care at nearby facilities [9].

Home births carry a higher risk of neonatal mortality compared with facility births in low- and middle-income countries [5]. Our analysis examines trends, determinants, and the associations of newborn care practices with newborn survival among home births in three South Asian countries. In Bangladesh, India, and Nepal, delivering at home is common. In Bangladesh as of 2014, 61% of births are delivered at home; in Nepal in 2016, 38% of births were home born. In India by 2015–16, home births constituted only 18% of births, representing a substantial decrease from 58% in 2005–06 (Additional file 1: Table S1). In these countries, we assess newborn care practices related to 1) thermal care: immediate drying and wrapping, delayed bathing, immediate skin-to-skin contact after home birth and 2) hygienic cord care: umbilical cord cutting with a clean instrument, and the absence of any harmful substance on the cord. This article is based on a technical report by the same authors [10], with additional and modified analyses to improve the study.

### Thermal care practices

Immediately drying the newborn, as recommended by the World Health Organization (WHO), has a number of benefits [11]. Immediately drying the newborn prevents the heat loss that occurs when amniotic fluid evaporates from an infant’s skin; babies born prematurely or underweight are particularly vulnerable to heat loss because of the large surface area of skin relative to their weight [12]. Hypothermia, even in warm climates, is a risk factor for newborn morbidity and mortality, although the contribution of hypothermia to neonatal mortality is poorly understood [12, 13]. Studies have shown that hypothermia was associated with an increase in neonatal mortality [14, 15]. In addition, cold stress is a risk factor for low blood sugar (hypoglycemia), as it renders the infant sleepy or irritable and unable to feed well, which further lowers blood sugar [16]. The act of drying may also help counteract birth asphyxia, via stimulation for infants experiencing difficulty breathing [17].

Drying and wrapping typically occur together [18]. Wrapping is also a relevant intervention for thermal care and is recommended in instances where the mother is unavailable for skin-to-skin contact [19, 20]. Although the importance of keeping babies warm is well understood, traditional beliefs or delivery in a home setting may interfere with a baby’s receipt of proper thermal care—cutting the cord and delivering the placenta may be seen as more urgent than drying the baby [21–23].

Early bathing also increases an infant’s risk of hypothermia. In addition, the vernix coating, the protective film that develops on the skin of the fetus that protects against infections, is washed away with early bathing. WHO recommends that bathing the child be delayed until after 24 h after birth, or at least for the first 6 h [24] yet bathing an infant soon after birth is a common practice around the world [25]. Beliefs around bathing include the belief that a newborn smells bad, that not bathing immediately will lead to body odor later in life, that the baby has a need to be clean or is dirty, that bathing will prevent infection, that visitors prefer clean babies, and that bathing will help to shape the baby’s head [23, 26].

Skin-to-skin contact between the baby and mother is recommended for the first hour of life after birth for any newborns without complications or that are low birthweight because it can help prevent hypothermia and encourage breastfeeding [27, 28]. Evidence has shown that kangaroo mother care, of which skin-to-skin contact is one of three essential components, considerably reduced neonatal mortality in preterm newborns among facility births [29].

### Hygienic cord care

Hygienic cord care, which includes cutting the cord with a new or sterilized instrument (or a clean delivery kit) as well as appropriate cord care [18], is a standard measure of newborn care [30]. Hygienic cord care is recommended to reduce the risk of sepsis, a major cause of newborn mortality—specifically, infection that enters the body at the cord stump site. Premature or low birthweight babies are at an increased risk of all-cause mortality. Their skin barrier function is compromised and their immune systems and vital organs may be underdeveloped; preterm babies may also lack or have reduced amounts of vernix, which is only developed in the later stages of pregnancy [13, 31, 32].

Although several studies have reported common usage of a clean instrument to cut the cord [18, 33–35], traditional practices of cutting with unclean objects are still found. For example, in Nepal the umbilical cord is sometimes cut against a rupee, called a “good luck coin” [36]. Other reports indicate that in Nepal and Bangladesh traditional practices include cutting the cord with household tools such a knife or a sickle; the instrument may have been placed on a dirty surface next to the woman [25, 37].

To reduce the risk of sepsis in places where home births are common and the neonatal mortality rate exceeds 30 deaths per 1000 live births, the WHO recommends applying 7.1% chlorhexidine digluconate solution or gel delivering 4% chlorhexidine daily to the cord stump during the first week of life [38]. The application of chlorhexidine to the cord as an alternative to the standard recommendation for dry cord care (applying nothing to the cord after it has been cut) is particularly relevant where harmful substances are traditionally placed on the cord, and it can serve as a safe substitute [24]. Successful trials in South Asia supported this practice [39–41]. Around the world, substances are placed on the cord stump to promote healing [26, 33]. In addition to hastening cord healing, traditional beliefs also include prevention of pain, infection, or bleeding, or to keep out evil spirits or cold air [33]. These substances have included powders, food, oils, herbs or spices, hot compresses, charcoal, antiseptics, tar, machine or motor oil, breastmilk, petroleum jelly, animal dung, among others [33]. The potential harm of these substances has not been entirely quantified, but the vulnerability of newborns to infection, especially sepsis, is well documented, and the cord stump provides a route for infection [33].

## Methods

### Aims

Interventions related to thermal care and clean cord care are evidence-based and low cost; however, there is scant research using population-based, nationally representative surveys to examine their coverage over time, examine the determinants of these practices, or examine how these practices may relate to neonatal mortality at national levels. The Demographic and Health Surveys (DHS) have included questions related to these practices in their household-based surveys for the past two decades. These questions have historically been asked of mothers who delivered at home, although more recent surveys in some countries have begun to assess some (but not all) of these practices among facility births [10]. While analysis of recent DHS surveys indicates that these interventions are often common practice in facility settings [10], they are also simple to implement among home deliveries and are universally recommended for newborn care [23].

In this study, we assessed home births in three countries where home delivery is common (see Additional file 1: Table S1), neonatal mortality holds the majority share of all under 5 deaths, and where data are available on newborn practices via multiple rounds of DHS surveys. While evidence of the importance of these interventions is well-documented, our paper seeks to understand the trends and predictors of these practices while also examining the evidence of the associations between these practices and home births at a population level in three countries. Thus, this paper addresses three questions:

1. Among home births in Bangladesh, India, and Nepal, how has coverage of these practices changed over time?
2. What are the key predictors of newborn care practices among home births in South Asia?
3. What are the associations between newborn care practices and newborn mortality among home births in South Asia?

### Data

This study used data from DHS surveys in 3 countries: Bangladesh (2007, 2011, 2014), India (2005–06, 2015–16), and Nepal (2006, 2011, 2016). The DHS Program conducted nationally representative, population-based household surveys in collaboration with the host countries. The surveys employed a multistage cluster sampling strategy. All women age 15–49 were eligible for interview in selected households. Mothers with a live birth in the 5 years preceding each survey (or 3 years in Bangladesh) received additional questions on care she received during pregnancy, birth, and in the postnatal period. These surveys include additional questions related to care of the baby immediately after delivery or in the first month of life. Our analysis included only women who delivered at home; Additional file 1: Table S1 includes the number of home births analyzed from each survey.

### Indicator construction

Table 1 shows the definitions of indicators of thermal care and cord care used in this analysis. We coded each variable into three categories: “yes” if the baby received the intervention as defined, “no” if the baby did not receive the intervention, or a third category for “don’t know” or missing responses. Drying and wrapping are assessed in one question in India, but with separate questions in Bangladesh and Nepal. Therefore, we coded drying and wrapping as one indicator. While chlorhexidine is the only recommended antiseptic to apply to the umbilical cord after it is cut, chlorhexidine use is only assessed in three surveys (Bangladesh 2014, Nepal 2011, and Nepal 2016). Further, as chlorhexidine use has only recently been promoted for use in high-mortality settings, implementation is still uncommon. Therefore, we grouped chlorhexidine with other antiseptics to create an “antiseptic” application category (see Table 1).

**Table 1:** Definitions of newborn care practices

Intervention	Indicator	WHO Recommendation^a^	Harmonized Response Categories	Notes
Thermal care	Immediate drying or wrapping	Dried or wrapped immediately^b^	(1) Dried or wrapped within 5 min of birth or before delivery of the placenta (2) Dried or wrapped after 5 min of birth or after delivery of the placenta (3) Not dried, don’t know, or missing	Only surveys in Bangladesh included options for not being dried or wrapped.
	Delayed bathing	Bathed after 24 h; however, after 6 h may be appropriate in certain contexts	(1) Bathed 6 h or more after birth (2) Bathed within the first 6 h of birth (3) Not bathed, don’t know, or missing	Question not included in India survey. Only surveys in Bangladesh included an option for “Not bathed”.
	Immediate skin-to-skin contact	Skin-to-skin contact between babies and mothers during the first hour after birth	(1) Skin-to-skin contact immediately after birth (2) No skin-to-skin immediately after birth (3) Don’t know, or missing	Question not included in India survey.
	Full thermal care	Immediate drying, delayed bathing, skin-to-skin	(1) All recommended thermal care interventions (2) None, some, don’t know, or missing	In India, the thermal care composite variable includes only the indicator for drying.
Hygienic cord care	Clean instrument used to cut the cord	A new or boiled instrument should be used to cut the cord	(1) A new or boiled instrument was used to cut the umbilical cord, or a clean delivery kit was used (2) A used or non-boiled instrument (3) Don’t know or missing	Clean instruments could include (boiled or new) blade, scissors, or knife. Other instruments included bamboo, sickle, or other.
	Nothing applied to the cord	Dry cord care is recommended; however, in high-mortality settings chlorhexidine is recommended	(1) Nothing was put on the umbilical cord stump (2) Only an antiseptic and no other substance (3) Any other substance applied (4) Don’t know or missing	Question not included in India survey. Antiseptics include chlorhexidine, methylated spirits, gentian violet, unspecified antibiotic. Other substances include mustard oil, ghee, turmeric, chewed rice, ginger juice, boric powder, talcum powder, vermillion, dung, local herbs.
	Full hygienic cord care	Clean instrument used to cut the cord and dry or antiseptic cord care	(1) Both recommended cord practices (2) Neither, only one, or don’t know or missing	In India, the cord care composite variable includes only the indicator for cord cutting.

^a^WHO 2017

^b^WHO 1997

We also created composite indicators of thermal care and hygienic cord care in order to assess predictors of receipt of care in Bangladesh, where many interventions were assessed in the survey. These variables comprised two categories: whether the newborn received all thermal care practices (immediate drying, delayed bathing, and skin-to-skin) or all clean cord care practices (clean instrument used to cut the cord and either dry cord care or antiseptic cord care), or whether they received some or none of the respective interventions, or had a “don’t know” or missing response.

### Analysis

#### Trends in newborn care practices

All analyses were performed with Stata v. 15 (StataCorp, College Station, TX). We first examined the extent to which babies born at home received each intervention over time in each country. “Don’t know” and missing responses were recoded into the “no” category for this section of the analysis. We tested the significance of the change between surveys by appending datasets within each country and applying bivariate logistic regression for each intervention.

#### Determinants of newborn care practices in South Asia

We next explored predictors of newborn care practices among home births in South Asia with data from the most recent surveys in Bangladesh, India, and Nepal using logistic regression. In India, the only two newborn care practices assessed were drying and the instrument used to cut the cord. For Nepal and Bangladesh, we explored predictors of composite indicators of thermal care and cord care, described in Table 1.

We explored several potential predictors of receipt of these recommended practices. In one study conducted in the same three countries (Bangladesh, India, and Nepal), maternal age, education, attendance at antenatal care (ANC), skilled attendance at birth, and gestational age of the baby were associated with use of a clean delivery kit in at least one of the three countries [42]. A study by Mullany et al. in 2010 [43] found an association between hypothermia and sex of the baby, thus warranting further study on newborn care practices by gender. We explored the above covariates as well as additional covariates of interest including place of residence (urban or rural), wealth quintile (recoded into three categories: lowest and second lowest, middle, and second highest and highest), and religion (Hindu and Muslim or other). We also examined preceding birth interval (first born, less than 2 years, and more than 2 years), and size at birth (as a proxy for gestational age, coded as small and normal or large, based on birth record or mother’s report in absence of the record).

#### Newborn care practices and newborn mortality

In addition to limiting our sample to home births in recent surveys—as there may be important confounders between mortality, facility delivery, and newborn practices—we also excluded infants who died immediately (on the first day of life). Structuring the analysis in this way avoided including cases where the death preceded an opportunity for implementation of these care practices although ensuring that the exposure to newborn care practices preceded the outcome (newborn mortality) was not possible for all our indicators. Drying/wrapping immediately, delayed bathing for at least 6 h, immediate skin-to-skin contact, and cutting the cord are events that occur on the first day, but the custom of putting a substance on the cord could occur later; the question on this indicator does not specify the exact timing. Finally, we excluded babies born in the most recent month before the survey to ensure that all births included in the sample had the potential to live through the entire newborn period.

We pooled data from the most recent surveys in Bangladesh (2014) and Nepal (2016) in order to increase the sample size and power to detect associations with newborn mortality. We analyzed data from India (2015–16) separately given large the discrepancies in the sample size and differences in questionnaires—Bangladesh and Nepal both included questions on bathing, skin-to-skin contact, and substance applied to the cord, whereas India did not. Data from Bangladesh and Nepal were weighted equally in the pooled sample. With these weights, the adjusted sample for Bangladesh 2014 and Nepal 2016 surveys included 4,214, with each survey accounting for half of the cases (2,107) in the pooled sample. After excluding births in the month preceding the survey and immediate newborn deaths, the reduced sample consisted of 4,115 births. In Nepal, we included all most recent births in the 5 years preceding the survey; Bangladesh’s survey only included births in the preceding 3 years. The sample for India included 34,324 births in 2015–16; after applying the restrictions noted, the sample reduced to 33,733.

Adjusted models controlled for known predictors of newborn mortality, including mother’s sociodemographic characteristics, care-related and care-seeking behaviors, and birth characteristics. Sociodemographic characteristics of the mother that have been found to be associated with newborn mortality are: place of residence, wealth, education, religion [44], maternal age at birth [45, 46], preceding birth interval [47], previous child under age 5 died [8], receipt of ANC [48], tetanus toxoid vaccine coverage [19], size at birth as a proxy for premature birth [46], gender of child, skilled attendance at birth, and postnatal care (PNC) [49]. Tetanus was included only in the adjusted models in India since it was not assessed in the Bangladesh 2014 survey. We did not include birth order because of its correlation with maternal age and preceding birth interval. We did not control for additional country-specific variables such as caste, region, or state. Each of those variables comprise many categories. Given the rarity of neonatal mortality, including those variables in an adjusted model would result in empty cells that would invalidate the model and regrouping those variables into fewer categories could negate their meaningfulness. For each independent variable, we selected the reference category as the category with the largest proportion.

For all adjusted, multivariable logistic regression models, we presented the area under the receiver operating characteristic curve (AUC), which reflects the predictive ability, or discriminatory ability, of the model, in a range from 0 to 1, where values closer to 1 indicate the best predictive ability and values closer to 0.5 indicate the modeling is no better than random chance. We also present the Pseudo R squared, interpreted as the proportion of the total deviance or variation in the dependent variable (mortality) that can be explained by the covariates in the model.

Unless otherwise specified, estimates are weighted using either survey weights or pooled weights. Analyses account for the complex sample design with adjustments for cluster and stratification.

## Results

### Trends

**Fig. 1:**
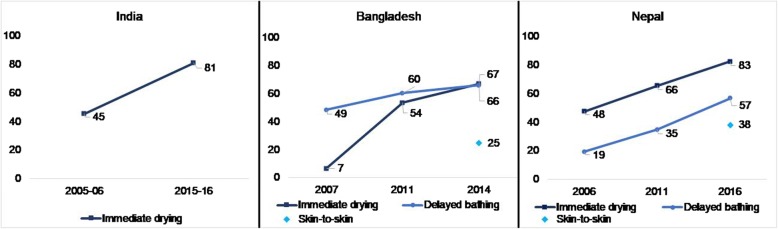
Coverage and trends in thermal care interventions. Notes: A solid line indicates a significant change (*p*-value < 0.05) between successive surveys, while a dotted line indicates no significant change. All differences between the oldest and the most recent survey were significant

Figure 1 demonstrates the change in thermal care practices in India, Bangladesh, and Nepal. Additional file 1: Table S2 contains these estimates and corresponding confidence intervals. India only assessed drying in each survey; between 2005 and 06 and 2015–16 the practice increased by nearly 40 percentage points to 81% in 2015–16. Immediate drying increased markedly in Bangladesh, from only 7% in 2007 to 66% in 2014, and in Nepal from 48 to 83% from 2006 to 2016. Delayed bathing increased nearly 20 percentage points in Bangladesh, reaching 66% in 2014, and over 40 percentage points in Nepal, reaching 57% in 2016. We tested the significance of the change between each survey and found significant changes between each survey for each country. Skin-to-skin contact, as assessed in recent surveys in Bangladesh and Nepal, was not common practice, only 25 and 38%, respectively.

**Fig. 2:**
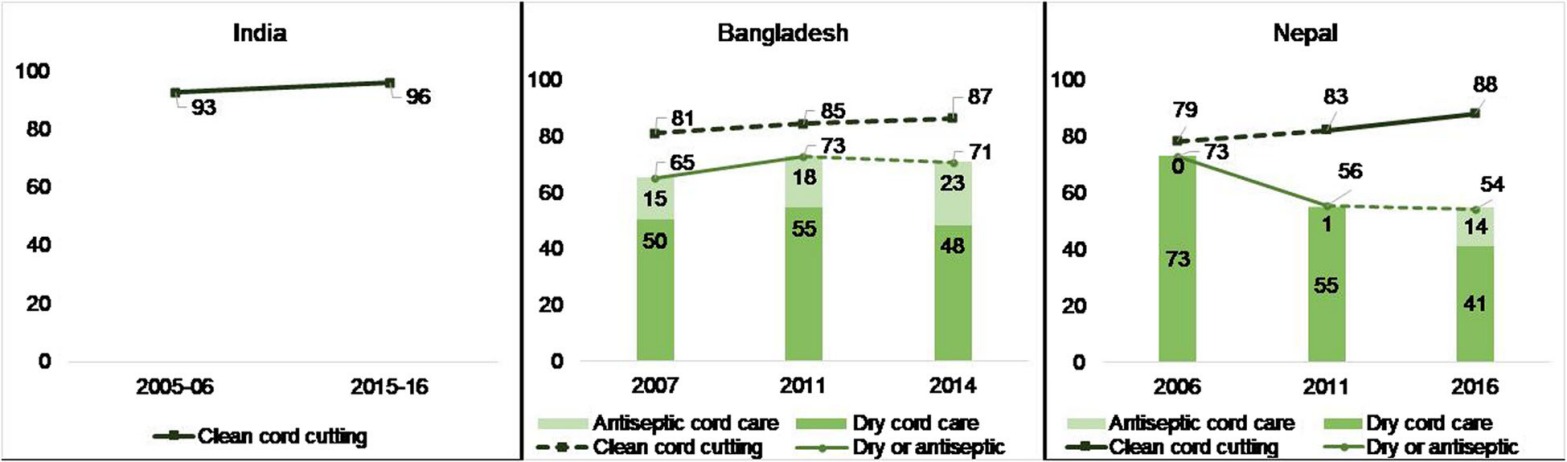
Coverage and trends in hygienic cord care interventions. Notes: A solid line indicates a significant change (*p*-value < 0.05) between successive surveys, while a dotted line indicates no significant change. All differences between the oldest and the most recent survey were significant

Figure 2 shows the percent of babies with clean cord care over time. Less substantial improvements occurred in the percent of babies born at home whose cord was cut with a clean instrument in each country; nonetheless, the changes were still significant. We observed minor but significant changes in India, from 93 to 96%, from 81 to 87% in Bangladesh, and from to 79 to 88% in Nepal. Figure 2 also shows the percentage of babies who had either no substance or an antiseptic placed on their cord. Additional file 1: Table S2 shows that chlorhexidine was the most common antiseptic in Nepal and an unspecified antibiotic and antiseptic were most common in Bangladesh. In Bangladesh, having either dry cord care and antiseptic cord care increased significantly over time, from 65% in 2007 to 71% in 2014, with minimal and non-significant change seen between 2011 and 2014. In Nepal, however, the combined dry or antiseptic care decreased significantly, from 73% in 2006 to 54% in 2016, despite an increase in antiseptic use from 1 to 14% between 2011 and 2016. This indicates that the application of substances other than antiseptics increased over time.

### Background characteristics

Table 2 shows the distribution of mother-baby demographic, socioeconomic, and care-seeking characteristics in Bangladesh, India, and Nepal, revealing many socioeconomic and health care access-related disadvantages among home births. In each country, the majority of home births occurred in rural residence. Mothers belonged to the poorest two wealth quintiles, had fewer than four ANC visits, and did not have a skilled attendant at birth. Most babies did not receive PNC within 2 days of delivery. In each country, there were more boys born alive than girls. The majority of home births in India and Nepal were fully protected against tetanus; tetanus was not assessed in the 2014 Bangladesh survey.

**Table 2:** Distribution of background characteristics among children born at home in the last 5 years

	India 2015–16		Bangladesh 2014		Nepal 2016	
	%	N	%	N	%	N
Place of residence						
Urban	15.2	5230	17.7	501	40.5	617
Rural	84.8	29095	82.3	2335	59.5	905
Wealth quintile						
Lowest and second lowest	74.4	25538	53.2	1509	59.9	912
Middle	14.0	4812	20.2	573	21.4	326
Second highest and highest	11.6	3974	26.6	754	18.7	284
Education						
None	53.7	18448	19.4	549	49.6	754
Primary	17.6	6038	33.3	945	23.6	359
Secondary or higher	28.7	9838	47.3	1341	26.8	408
Religion						
Hindu	71.3	24484	5.5	156	84.9	1,292
Muslim, other	28.7	9841	94.5	2680	15.1	230
Maternal age at birth						
< 18	2.2	772	16	454	5.1	78
18–34	89.2	30601	79.6	2259	88.4	1,345
35 and older	8.6	2952	4.3	123	6.5	98
Preceding birth interval						
First birth	16.0	5499	33.7	955	19	289
Less than 2 years	22.3	7644	8.4	237	17.7	269
2 years or more	61.7	21182	58	1644	63.3	964
Previous child under 5 die						
No	84.4	28978	88.1	2498	86.2	1,311
Yes	15.6	5347	11.9	338	13.8	210
Antenatal care						
Less than 4 visits	76.2	26166	79.1	2243	51.6	785
4 or more visits	23.8	8159	20.9	593	48.4	736
Tetanus^a^						
Not fully protected	18.9	6476	n/a		17.1	260
Fully protected	81.1	27849			82.9	1,261
Size at birth						
Normal or Large	82.3	28234	78.9	2236	82.7	1,258
Small or very small	17.7	6090	21.1	600	17.3	264
Gender of child						
Female	46.5	15956	48.2	1368	45.3	690
Male	53.5	18369	51.8	1468	54.7	832
Skilled attendant at birth						
No	78.3	26873	91.8	2602	93.1	1,417
Yes	21.7	7451	8.2	233	6.9	105
PNC in 2 days						
No	83.3	28578	61.4	1740	87.2	1,327
Yes	16.7	5747	38.6	1096	12.8	194
Died the first 28 days						
No	97.5	33453	98.1	2783	98.7	1,501
Yes	2.5	871	1.9	53	1.3	20
Total	100.0	34324	100.0	2836	100.0	1,521
Mortality analysis						
	India 2015–16		Bangladesh 2014 and Nepal 2016			
Number of excluded cases^b^	592		99			
Number of deaths on days 1–28	555		34			
Total for mortality analysis	33733		4115			

^a^Tetanus was not assessed in the Bangladesh 2014 survey

^b^Excluded cases included births occurring in the 28 days preceding the survey and newborn deaths occurring on the day of birth

### Predictors of newborn care practices in South Asia

We examined the predictors of newborn care interventions in the most recent surveys in India, Bangladesh, and Nepal. Tables 3 and 4 present the results of the logistic regressions. We conducted separate models for the indicators of thermal care (drying in India, full thermal care in Bangladesh and Nepal) and cord care (cutting with a clean instrument in India, full hygienic cord care in Bangladesh and Nepal). No one covariate significantly predicted receipt of thermal care interventions across all three countries, although some predicted care in two of the three countries (Table 3). For example, babies born to mothers with secondary or higher education in India (Adjusted Odds Ratio (AOR) AOR = 1.1, 95% Confidence Interval (CI): 1.0–1.2) and Bangladesh (AOR = 1.6, CI: 1.0–2.5) were more likely to receive thermal care practices than babies born to mothers with no education. In Nepal, babies born to mothers in wealthier quintiles have over two times the odds of receiving thermal practices compared with lower wealth quintiles (Second highest and highest wealth, AOR = 2.3, CI: 1.6–3.4). In India, babies born into the middle wealth quintile were less likely to be immediately dried compared with lower wealth quintiles (AOR = 0.8, CI: 0.7–0.9). In both India and Nepal, we found that having an SBA was positively and significantly associated with a baby’s receipt of the recommended thermal care interventions after controlling for other variables. Babies delivered by a skilled attendant had at least one and one-half times the odds receiving both thermal care practices compared with those who were delivered without an SBA (India: AOR = 1.4, CI: 1.3–1.6; Nepal: AOR = 2.4, CI: 1.4–4.2). In India as well, a mother’s receipt of ANC also predicted the baby would be dried immediately (AOR = 1.4, CI: 1.2–1.5).

**Table 3:** Adjusted odds of receiving newborn care interventions among most recent home births in India, Bangladesh, and Nepal

	India 2015–16 (immediate drying)				Bangladesh 2014 (immediate drying, delayed bathing, and skin-to-skin)				Nepal 2016 (immediate drying, delayed bathing, and skin-to-skin)			
	UOR	95% CI	AOR	95% CI	UOR	95% CI	AOR	95% CI	UOR	95% CI	AOR	95% CI
Place of residence (ref = rural)												
Urban	0.9*	[0.8,1.0]	0.9*	[0.8, 1.0]	0.7	[0.5,1.0]	0.6*	[0.4, 0.9]	0.7	[0.5,1.1]	0.7	[0.5, 1.1]
Wealth quintile (ref = lowest and second)												
Middle	0.8**	[0.7,0.9]	0.8***	[0.7, 0.9]	1.1	[0.7,1.6]	1.0	[0.6, 1.5]	2.4***	[1.7,3.4]	2.2***	[1.5, 3.1]
Second highest and highest	1.1	[1.0,1.2]	1.0	[0.9, 1.2]	1.4	[1.0,1.9]	1.4	[1.0, 2.0]	2.4***	[1.6,3.4]	2.3***	[1.6, 3.4]
Education (ref = none)												
Primary	1.2**	[1.1,1.3]	1.1*	[1.0, 1.3]	1.2	[0.8,1.8]	1.3	[0.8, 1.9]	0.8	[0.6,1.2]	0.8	[0.6, 1.2]
Secondary or higher	1.2***	[1.1,1.3]	1.1**	[1.0, 1.2]	1.7*	[1.1,2.7]	1.6*	[1.0, 2.5]	0.8	[0.6,1.2]	0.8	[0.6, 1.2]
Religion												
Hindu	ref				0.7	[0.4, 1.3]	0.8	[0.4, 1.5]	ref			
Muslim, other	0.9*	[0.8,1.0]	0.9	[0.8, 1.0]	ref				1.1	[0.6,2.2]	1.0	[0.6, 1.8]
Maternal age at birth (ref = 18–34)												
< 18	1.0	[0.7,1.3]	1.0	[0.7, 1.3]	1.0	[0.7,1.5]	1.0	[0.6, 1.6]	0.8	[0.5,1.4]	1.0	[0.5, 1.8]
35 and older	0.9*	[0.8,1.0]	0.9	[0.8, 1.0]	0.8	[0.4,1.7]	0.9	[0.4, 1.9]	0.7	[0.4,1.4]	0.9	[0.5, 1.5]
Preceding birth interval (ref = 2+)												
First birth	1.0	[0.9,1.1]	1.0	[0.9, 1.1]	1.0	[0.7,1.4]	0.9	[0.6, 1.3]	1.1	[0.7,1.5]	1.1	[0.7, 1.7]
Less than 2 years	1.0	[0.9,1.1]	1.0	[0.9, 1.1]	0.8	[0.4,1.4]	0.8	[0.4, 1.4]	1.7**	[1.2,2.5]	1.6*	[1.1, 2.4]
Antenatal care (ref = less than 4)												
4 or more visits	1.4***	[1.3,1.6]	1.4***	[1.2, 1.5]	1.3	[0.9,1.8]	1.2	[0.8, 1.7]	0.9	[0.6,1.2]	0.9	[0.7, 1.3]
Size at birth(ref = normal or large)												
Small or very small	0.9	[0.9,1.0]	0.9	[0.8, 1.0]	1.7**	[1.2,2.3]	1.8***	[1.3, 2.5]	1.2	[0.8,1.7]	1.3	[0.9, 1.8]
Gender of child (ref = female)												
Male	1.0	[0.9,1.0]	1.0	[0.9, 1.0]	1.0	[0.8,1.3]	1.0	[0.8, 1.3]	0.9	[0.7,1.1]	0.8	[0.7, 1.1]
Skilled attendant at birth (ref = no)												
Yes	1.5***	[1.4,1.7]	1.4***	[1.3, 1.6]	1.5	[1.0,2.5]	1.4	[0.9, 2.4]	2.8***	[1.7,4.6]	2.4**	[1.4, 4.2]
ROC			0.57				0.59				0.64	
Pseudo R2			0.01				0.01				0.04

**Table 4:** Adjusted odds of receiving newborn care interventions among most recent home births in India, Bangladesh, and Nepal

	India 2015–16 (clean instrument to cut the cord)				Bangladesh 2014 (clean instrument, dry or antiseptic cord care)				Nepal 2016 (clean instrument, dry or antiseptic cord care)			
	UOR	95% CI	AOR	95% CI	UOR	95% CI	AOR	95% CI	UOR	95% CI	AOR	95% CI
Place of residence (ref = rural)												
Urban	1.2	[0.9,1.6]	1.1	[0.8, 1.5]	1.0	[0.7,1.3]	1.0	[0.8, 1.4]	1.1	[0.8,1.5]	1.1	[0.8, 1.4]
Wealth quintile (ref = lowest and second)												
Middle	1.0	[0.8,1.3]	0.9	[0.7, 1.1]	1.1	[0.7,1.6]	1.0	[0.7, 1.4]	0.7*	[0.5,1.0]	0.8	[0.6, 1.0]
Second highest and highest	1.4*	[1.0,1.9]	1.2	[0.8, 1.6]	1.0	[0.7,1.3]	0.8	[0.6, 1.2]	0.8	[0.6,1.1]	0.8	[0.6, 1.1]
Education (ref = none)												
Primary	1.1	[0.9,1.3]	1.1	[0.9, 1.3]	0.9	[0.7,1.2]	1.0	[0.8, 1.3]	1.1	[0.8,1.4]	1.1	[0.8, 1.4]
Secondary or higher	1.3**	[1.1,1.6]	1.3**	[1.1, 1.6]	1.2	[0.9,1.7]	1.3	[1.0, 1.7]	1.6**	[1.2,2.1]	1.5*	[1.1, 2.1]
Religion												
Hindu	(ref)				1.3	[0.8, 1.9]	1.2	[0.8, 1.9]	(ref)			
Muslim, other	0.9	[0.8,1.1]	0.9	[0.8, 1.1]	(ref)				0.9	[0.6,1.3]	1.0	[0.6, 1.5]
Maternal age at birth (ref = 18–34)												
< 18	0.7	[0.5,1.0]	0.8	[0.5, 1.2]	0.8	[0.6,1.0]	1.0	[0.7, 1.4]	1.0	[0.6,1.6]	0.8	[0.5, 1.4]
35 and older	0.7***	[0.6,0.8]	0.7**	[0.6, 0.9]	1.0	[0.6,1.5]	1.1	[0.7, 1.8]	0.8	[0.5,1.3]	0.9	[0.6, 1.4]
Preceding birth interval (ref = 2+)												
First birth	0.8	[0.7,1.0]	0.7**	[0.6, 0.9]	0.7	[0.5,1.1]	0.7	[0.4, 1.1]	1.1	[0.8,1.5]	1.0	[0.7, 1.4]
Less than 2 years	0.9	[0.8,1.1]	0.9	[0.8, 1.1]	1.5*	[1.0,2.2]	1.4	[1.0, 2.1]	0.9	[0.7,1.2]	0.9	[0.7, 1.2]
Antenatal care (ref = less than 4)												
4 or more visits	1.3**	[1.1,1.6]	1.3*	[1.0, 1.5]	1.5*	[1.1,2.2]	1.5**	[1.1, 2.0]	1.2	[0.9,1.5]	1.1	[0.8, 1.3]
Size at birth (ref = normal or large)												
Small or very small	1.1	[0.9,1.3]	1.0	[0.9, 1.2]	0.8	[0.6,1.1]	0.9	[0.7, 1.1]	1.0	[0.8,1.4]	1.0	[0.8, 1.4]
Gender of child (ref = female)												
Male	1.0	[0.9,1.1]	1.0	[0.8, 1.1]	0.8	[0.7,1.0]	0.8	[0.7, 1.0]	0.8	[0.7,1.0]	0.8	[0.7, 1.0]
Skilled attendant at birth (ref = no)												
Yes	1.1	[0.9,1.3]	1.0	[0.8, 1.2]	1.7**	[1.2,2.5]	1.7*	[1.1, 2.4]	1.0	[0.7,1.6]	1.0	[0.7, 1.6]
ROC			0.65				0.58				0.57	
Pseudo R2			0.04				0.02				0.01	

In the adjusted models examining hygienic cord practice (Table 4), care before and during birth was associated with receipt of both recommended cord care practices. Mothers' attendance at four or more ANC visits was associated with increased odds of hygienic cord care by 30% compared with no ANC in India (AOR = 1.3, CI: 1.0–1.5) and by 50% in Bangladesh (AOR = 1.5, CI: 1.1–2.0). Having an SBA at delivery in Bangladesh was associated with a 70% increase in the odds compared with having no SBA (AOR = 1.7, CI: 1.1–2.4). In India and Nepal, maternal secondary or higher education was associated with an increased likelihood of babies receiving hygienic cord care.

Low birthweight or premature babies are most in need of these recommended thermal and hygienic cord care practices, and small babies in Bangladesh were more likely to be dried immediately, have delayed bathing, and receive skin-to-skin compared with normal or large sized babies (AOR = 1.8, CI: 1.3–2.5). Small babies were not more likely to receive clean cord care interventions in any country. Gender of the child and religion of the mother also did not significantly predict receipt of these interventions in any country.

All of the models examining receipt of recommended practices demonstrated poor, if not failing, discriminatory ability; the AUC ranged from 0.57 to 0.65. The models explained only 1 to 4% of the deviance in thermal care or hygienic cord care. These fit statistics indicate that our models omit other factors of relative importance to receipt of care; however, across the interventions, we found a pattern of significant associations between care during pregnancy and birth, and receipt of newborn care practices.

### Associations between newborn care practices and mortality

Table 5 presents the unadjusted and adjusted odds ratios of newborn death on days 1–28 after birth by newborn care practices for India and Bangladesh and Nepal, respectively. In India, we found that babies whose cord was cut with an unclean instrument had 1.6 times the odds of dying compared with babies whose cord was cut with a clean instrument or a blade from a kit (*p*-value < 0.05) although in the adjusted model, the odds were reduced (AOR = 1.4) and the association became non-significant. It should be noted that “don’t know” was not a response option for this question, which may have affected these findings. Immediately drying and wrapping the baby was not significantly associated with newborn mortality in the multivariable regression analyses. We also found that firstborn children (AOR = 2.6, CI: 1.9–3.5) and children born fewer than 2 years after their mother’s last child (AOR = 1.4, CI: 1.1–1.9) had increased odds of dying compared with babies born to mothers with a longer birth interval (two or more years). Babies born to mothers over age 35 (compared with age 18–34) or who had a previous child die before age 5 (compared with no previous child death), who were not protected from tetanus, who were small at birth, and who had no PNC checkup in 2 days (compared to those without a check) were less likely to survive. Babies who did not receive a PNC checkup had a twofold increase in the odds of dying compared with those who were checked (AOR = 2.1, CI: 1.5–3.1).

**Table 5:** Unadjusted and Adjusted Odds Ratios of the association with mother, child and care characteristics and death of the newborn during days 1–28 after birth

	India 2015–16				Bangladesh 2014 and Nepal 2016			
	UOR	95% CI	AOR	95% CI	UOR	95% CI	AOR	95% CI
Country (ref = Nepal)								
Bangladesh	n/a		n/a		1.8	0.8, 4.1	2.3	1.0, 5.2
Place of residence (ref = rural)								
Urban	1.1	0.7, 1.5	1.3	0.9, 1.8	0.6	0.2, 1.4	0.7	0.3, 1.7
Wealth quintile (ref = lowest and second)								
Middle	1.2	0.9, 1.7	1.3	1.0, 1.8	0.1**	0.0, 0.4	0.1**	0.0, 0.5
Second highest and highest	0.7	0.4, 1.0	0.8	0.5, 1.3	0.9	0.4, 2.4	1.3	0.4, 4.1
Education (ref = none)								
Primary	1.2	0.9, 1.6	1.4*	1.1, 1.8	1.5	0.5, 4.1	1.4	0.5, 4.0
Secondary or higher	0.9	0.7, 1.2	1.0	0.7, 1.4	1.4	0.5, 3.7	1.9	0.6, 5.6
Religion (ref = Hindu)								
Muslim, other	0.7*	0.6, 0.9	0.7**	0.5, 0.9	1.3	0.6, 2.8	0.4	0.2, 1.0
Maternal age at birth (ref = 18–34)								
< 18	1.5	0.8, 2.6	1.0	0.5, 1.8	2.1	0.9, 5.2	2.6	1.0, 7.0
35 and older	1.9***	1.4, 2.4	1.8***	1.4, 2.5	1.7	0.3, 8.5	1.6	0.4, 6.7
Preceding birth interval (ref = 2+)								
First birth	1.8***	1.4, 2.4	2.6***	1.9, 3.5	1.0	0.5, 2.2	0.6	0.3, 1.3
Less than 2 years	1.4**	1.1, 1.9	1.4*	1.1, 1.9	0.5	0.1, 2.3	0.5	0.1, 2.3
Previous child under 5 die (ref = no)								
Yes	2.3***	1.8, 2.8	2.4***	1.9, 3.1	1.1	0.4, 3.2	1.1	0.4, 2.9
Antenatal care (ref = less than 4)								
4 or more visits	0.9	0.6, 1.2	1.0	0.8, 1.4	0.2*	0.0, 0.9	0.2*	0.1, 0.9
Tetanus (ref = fully protected)								
Not fully protected	1.5**	1.2, 1.9	1.3*	1.0, 1.7	n/a			
Size at birth (ref = normal or large)								
Small or very small	1.9***	1.5, 2.5	1.9***	1.4, 2.5	1.7	0.7, 3.8	1.3	0.5, 3.1
Gender of child (ref = female)								
Male	1.1	0.9, 1.3	1.1	0.9, 1.4	0.6	0.3, 1.4	0.7	0.3, 1.5
Skilled attendant at birth (ref = no)								
Yes	0.7*	0.5, 1.0	0.8	0.6, 1.1	0.1*	0.0, 0.9	0.1*	0.0, 0.9
PNC in 2 days (ref = yes)								
No	2.2***	1.5, 3.2	2.1***	1.5, 3.1	1.5	0.6, 3.9	1.4	0.5, 3.8
Dry or wrapping (ref = immediately)								
Delayed	1.2	0.9, 1.6	1.1	0.8, 1.5	0.7	0.3, 2.0	0.6	0.2, 1.8
Not dried, don’t know, missing	n/a		n/a		1.3	0.3, 6.0	0.9	0.2, 4.3
Bathing (ref = delayed)								
Immediately	n/a		n/a		1.4	0.6, 3.3		
Not bathed, don’t know, missing					52.4***	18.2,150.6		
Skin to skin (ref = yes)								
No*	n/a				1.0	0.4, 2.2	0.7	0.3, 1.7
Instrument used to cut the cord (ref = sterile)								
Unclean	1.6*	1.0, 2.7	1.4	0.8, 2.4	2.4*	1.1, 5.4	2.0	0.9, 4.3
Don’t know	n/a				1.4	0.2, 10.5	1.7	0.2, 17.7
Substance put on the stump (ref = nothing)								
Antiseptic only	n/a				0.1**	0.0, 0.4	0.2**	0.0, 0.6
Other	n/a				0.6	0.3, 1.5	0.7	0.3, 1.6
Don’t know or missing	n/a				1.7	0.5, 5.3	1.8	0.5, 6.5
ROC			0.68				0.77	
Pseudo R2			0.04				0.09	

In the pooled sample of births in Bangladesh and Nepal, the multivariable logistic regression results indicated significant associations between cord care practices and newborn death. Babies who had an antiseptic applied to the cord were less likely to die than babies with dry cord care (AOR = 0.2, CI: 0.0–0.6). In an unadjusted model, babies whose cord was cut with an unclean instrument had almost two and a half times the odds of dying compared with babies with a clean instrument used (Unadjusted Odds Ratio (UOR) = 2.4, CI: 1.1–5.4). However, when controlling for other covariates in the adjusted model, the odds were reduced and the association became non-significant (AOR = 2.0, CI: 0.9–4.3).

The unadjusted regression analysis revealed alarmingly strong associations between newborn death and the “don’t know” or missing responses to questions on bathing in Bangladesh and Nepal. Babies whose mothers did not provide a valid response about bathing had over 50 times the odds of dying compared with mothers who said their baby was bathed at least 6 h after birth (UOR = 52.4, CI: 18.2–150.6). Eight of the 32 unweighted cases with missing or “don’t know” responses referred to newborns who had died after the first day. While of course the absence of a response to the survey does not cause mortality, mortality could lead to the absence of a response, a phenomenon we describe in the discussion of this paper. Thus, given the nature of the relationship, we did not include the bathing or combined thermal care variables in our adjusted models.

Very few covariates significantly predicted newborn mortality in Bangladesh and Nepal; among them were attendance at ANC and SBA. Mothers attending four or more ANC visits compared with fewer than four visits (AOR = 0.2, CI: 0.1–0.9) and mothers delivering with a SBA compared with a traditional attendant or no attendant (AOR = 0.1, CI: 0.0–0.9) were significantly less likely to die on days 1–28. Although there may be a potential interaction between skilled birth attendance and receipt of recommended practices, the frequencies of mortality were too small for identification of an interaction effect.

When comparing model fit statistics among all the models in India, and Bangladesh and Nepal, we found fair discriminatory ability in the pooled sample of Bangladesh and Nepal surveys (AUC = 0.77). The model in India resulted in an AUC of 0.68, or poor discrimination. The Pseudo R^2^ estimates were also highest for models conducted with recent Bangladesh and Nepal survey data, and indicate 9% of the deviance in mortality is explained by the covariates in the model, while in India, the model predicted 4% of the deviance in mortality. As stated earlier, these models cannot account for all potential causes of mortality; other omitted factors for which we could not control likely contribute to newborn mortality, for example, congenital defects or intrapartum complications.

## Discussion

### Overview of findings

This study reviewed coverage of recommended newborn practices—immediate drying, delayed bathing, skin-to-skin contact, clean instruments used to cut the umbilical cord, and hygienic cord care—over time among home births in Bangladesh, India, and Nepal. We also examined predictors of newborn care practices in those countries. We further explored associations between recommended newborn care practices and neonatal mortality for babies born in a home setting, pooling data from Bangladesh and Nepal to increase the sample size.

Thermal care practices including immediate drying and delayed bathing showed more substantial increases in use between the earlier and later survey periods compared with the hygienic cord care indicators (clean instrument used to cut the cord and either dry or antiseptic cord care) although clean cord-cutting instruments were common even in earlier surveys. Antiseptic application increased among home births in Nepal and Bangladesh. However, in Nepal, there was also an increase in the application of substances other than antiseptics. Coffey and Brown (2017) and others noted the importance of cultural practices regarding care for the umbilical cords that the desire to care for the cord with topical application of substances persists across cultural contexts; there is also a paucity of research demonstrating that all traditional substances are harmful [33, 50].

We found that SBA and attendance at ANC often positively predicted receipt of recommended thermal and hygienic cord care practices. While low birthweight or premature babies may be most susceptible to the consequences of a lack of thermal care or unhygienic cord practices, our study showed that in most countries and for most interventions, these babies do not receive the interventions any more than normal or larger birthweight babies, except for small babies in Bangladesh. Neither sex of the baby nor religion predicted receipt of care.

After controlling for several known predictors of newborn mortality using survey data from South Asia, we found significant associations between neonatal mortality and cord care, though not thermal care. In India and in a pooled sample of births from Bangladesh in Nepal, cutting the cord with a clean instrument was associated with newborn survival though only in bivariate regressions. In a pooled analysis of three randomized control trials examining the relationship between sepsis-related newborn mortality and clean cord care practices in Bangladesh, India, and Nepal, there was a significant reduction in mortality among births where a clean birth kit was used, in addition to handwashing, use of a boiled blade, boiled thread, and antiseptic cord care [42]. Similarly, in our multivariable analysis in Bangladesh and Nepal, we found that application of antiseptics (compared with dry cord care) revealed a large reduction in the odds of dying on days 1–28 after birth. Seward et al. found higher odds of dying among babies with dry cord care in Bangladesh, but lower odds in Nepal [42]. Randomized, controlled trials have indicated the effectiveness of chlorhexidine in reducing mortality [39, 40].

ANC provides an opportunity to counsel mothers about recommended newborn care practices and to encourage skilled attendance at birth. Among home births, we found that skilled care before and during birth leads to better health practices, and that newborns who receive recommended newborn care practices—specifically, hygienic cord care—along with antenatal, intrapartum, and PNC have lower odds of dying. These interventions have also been widely recommended to promote better newborn care practices [23, 51–54]. Given these findings, promotion of both use of maternal health services and the recommended newborn practices may have synergistic effects on newborn survival.

### Strengths, limitations, and recommendations

Strengths of this study included the ability to gain more power by pooling data from two geographically proximate countries—Nepal and Bangladesh—where the most recent DHS surveys were conducted within a two-year period and contained similar questions. Second, by restricting our sample to home births, excluding deaths in the first day (when most newborn deaths occur [55]), and excluding births in the most recent month, we could better assure that the newborn care interventions (except substance applied to the cord) occurred prior to death. Although this study cannot indicate the causality of relationships, addressing temporality in our analysis in this way greatly reduces the chance of a reverse causal relationship. Nonetheless, as drying a baby immediately may also help a baby breathe in addition to providing thermal support, by excluding deaths on the day of birth, we may underestimate the relationship between drying and survival.

There are limitations to this analysis. Even though pooling data from Bangladesh and Nepal increased the total sample size, the pooled sample included only 39 deaths (unweighted). Although the entire set of questions related to these practices are only consistently asked of home births, limiting our study to this population reduced our sample size and the power to detect significant associations. Additionally, there are many causes of newborn mortality that the interventions examined in this study are not expected to prevent; for example, congenital factors or intrapartum birth complications. Our analysis does not control for all potential contributing factors to mortality. Our indicators, particularly for hygienic cord care, may lack critical information. In order to ensure clean cord care, not only must a clean object be used to cut the cord, but any other object that comes in contact with the cord must also be clean, including hands, clamps, towels, or the surface on which the cord is cut. While an instrument may have been boiled before cutting the cord, we cannot know if it was done properly and if it remained clean until use, especially in a home birth setting. Hygienic cord care programs should promote all areas of sanitary concern and can be done in a way that honors tradition. For example, the Ministry of Health and Nepal promoted the use of a clean delivery kit that was produced by the Maternal and Child Health Products Pvt. Ltd. in Nepal with assistance from PATH. The birth kits include a sterile, plastic “good luck” coin to replace the practice of using a dirty coin as a surface against which the cord can be cut [25, 36]. Kits may also include clean instruments, soap, a clean sheet on which a mother can deliver, clean string to tie the cord, and instructions for the birth attendant or mother [42].

Although chlorhexidine is the only recommended antiseptic for use on the cord stump, our definition of antiseptic included other substances, the efficacy of which have not all been studied [33]. Products could be placed on the cord at any time before the stump heals. Thus, it is plausible that our finding that antiseptic care is protective against death over dry cord care could reflect not that dry cord care is harmful, but that babies died before caregivers had a chance to apply anything. On the other hand, if mothers knew that placing any substance on the cord may be harmful, this question is susceptible to social desirability bias in that mothers do not report other practices. It is also possible that mothers did not understand the terms antiseptic or chlorhexidine, leading to inaccurate reporting of antiseptic application. In the recent Nepal 2016 DHS, the local brand name Navi Malam was used in the questionnaire, which hopefully mitigated reporting bias. Given these potential biases and limitations, the conclusion that both other substances and antiseptics are protective against death compared with dry cord care should be regarded with caution.

Finally, we cannot draw direct comparisons between findings in India and neighboring countries Bangladesh and Nepal. The extent to which and the reasons why women deliver at home versus at a facility differ among the countries. The survey in India included only two questions related to thermal care and cord care; whereas, in Bangladesh and Nepal, several other questions were included. Additionally, the India survey contained no missing or “don’t know” responses from mothers regarding newborn practices due to an error in the data collection instrument. Although the questionnaire included a response option for “don’t know”, the Computer-Assisted Personal Interviewing program erroneously omitted this option in the India survey.

### Validity and recall of newborn care practices

Death of a newborn child is traumatic; recall around traumatic events has important implications for our study. Women who have lost a child are at risk of psychiatric disorders including depression, anxiety, or even posttraumatic stress disorder (PTSD) [56]. Memory of details around a traumatic event is compromised, and those who suffer from PTSD or related disorders are prone to reduced recall of some details; further, individuals who have experienced trauma may avoid discussing the event in detail as a means of emotional protection, known as “functional avoidance” [57, 58]. In some cultures, talking about a stillbirth or neonatal death may be discouraged [22], which in turn may lead to worse recall. The less often an event is recalled, the less likely it will be remembered. That we found such a strong association between the “don’t know” and missing responses about bathing and newborn death suggests that this survey question might be particularly sensitive to functional avoidance or recall errors, although further investigation is warranted. However, our findings elicit concerns about the validity of other responses related to newborn care by bereaved mothers. Based on these results, we recommend a review of compassionate ways to collect these data, particularly around sensitive issues such as care preceding the loss of a baby.

Even among mothers whose most recent child survived the first month, the validity of the responses to these questions is still uncertain. Studies show that mothers had difficulty relaying and recalling timing or sequence of the newborn care and postnatal care intervention [59–61]. A study in Kenya [62] showed that accuracy of reporting on newborn care practices was affected by wording of the question and context of the indicator; for example, a two-part question that explained skin-to-skin care notably lessened women over-reporting their receipt of this practice. The DHS includes a single question.

## Conclusion

Our study identifies substantial gains in thermal care practices and use of a clean instrument to cut the cord among babies born at home in Bangladesh, India, and Nepal. In Bangladesh and Nepal, although use of antiseptics including chlorhexidine have increased, traditional practices of placing other or potentially harmful substances on the umbilical cord stump persist. We found that care before and during birth was associated with an increased likelihood of implementation of these recommended practices even among home births; SBA, along with hygienic cord care, were also associated with reduced odds of newborn mortality. However, these findings should be interpreted with caution given the potential interference of bias and relatively few cases of deaths even in pooled samples. Finally, the significant association found between newborn mortality and missing or “don’t know” survey responses about newborn care may suggest that recall of details surrounding the traumatic event of a loss of a child is unreliable.

## Additional file

Additional file 1: **Table S1.** Most recent births in the 3 or 5 years preceding the survey analyzed in this report. **Table S2.** Percent of babies who received newborn care interventions among home births. **Table S3.** Antiseptic placed on the umbilical cord among home births. (DOCX 28 kb)

## Data Availability

All data are publicly available from https://dhsprogram.com/data/available-datasets.cfm.

## Abbreviations

ANC: Antenatal care
AOR: Adjusted odds ratio
AUC: Area under the receiver operating characteristic curve
CI: Confidence interval
DHS: Demographic and Health Surveys
PNC: Postnatal care
PTSD: Posttraumatic stress disorder
SBA: Skilled birth attendant
UOR: Unadjusted odds ratio
WHO: World Health Organization
