# Supplementary material for: Trends, determinants, and newborn mortality related to thermal care and umbilical cord care practices in South Asia
Source: BMC Pediatr. 2019 Jul 22;19:248. doi: 10.1186/s12887-019-1616-2 (PMC6647093; doi:10.1186/s12887-019-1616-2)
Supplement: Supplementary file 1 — Table S1. Most recent births in the 3 or 5 years preceding the survey analyzed in this report. Table S2. Percent of babies who received newborn care interventions among home births. Table S3. Antiseptic placed on the umbilical cord among home births. (DOCX 28 kb) [file 12887_2019_1616_MOESM1_ESM.docx]

**List of Supplemental Tables**

Supplemental Table 1. Most recent births in the 3 or 5 years preceding the survey analyzed in this report

Supplemental Table 2. Percent of babies who received newborn care interventions among home births

Supplemental Table 3. Antiseptic placed on the umbilical cord among home births

Table S1. Most recent births in the 3 or 5 years preceding the survey analyzed in this report

|  | **Women with a live birth** | **Children born at home** |
| --- | --- | --- |
|  | **N** | **N (%)** |
| Bangladesh 2007 | 3352 | 2752 (82.1) |
| Bangladesh 2011 | 4652 | 3291 (70.8) |
| Bangladesh 2014 | 4627 | 2836 (61.3) |
| India 2005-06 | 39677 | 23135 (58.3) |
| India 2015-16 | 184641 | 34325 (18.6) |
| Nepal 2006 | 4066 | 3221 (79.2) |
| Nepal 2011 | 4148 | 2480 (59.8) |
| Nepal 2016 | 3998 | 1521 (38.1) |
| Note: Ns and % based on most recent births born in the 5 years preceding each survey in India and Nepal. In Bangladesh, estimates are based on births in the preceding 3 years (2014 and 2011) or births that occurred between the date of the interview and January 2004 (2007). Unless specified, all counts are weighted using individual survey weights. | | |

|  | **Immediate drying** | | **Delayed bathing** | | **Skin-to-skin** | | **Full thermal care** | | **Clean cord cutting** | | **Dry or antiseptic cord care** | | **Full hygienic cord care** | | |
| --- | --- | --- | --- | --- | --- | --- | --- | --- | --- | --- | --- | --- | --- | --- | --- |
| **Survey** | **%** | **95% CI** | **%** | **95% CI** | **%** | **95% CI** | **%** | **95% CI** | **%** | **95% CI** | **%** | **95% CI** | **%** | **95% CI** |  |
| Bangladesh 2007 | 6.6 | [5.4,8.1] | 48.5 | [45.6,51.5] |  |  |  |  | 81.3 | [78.3,84.0] | 65.4 | [65.5, 68.1] |  |  |  |
| Bangladesh 2011 | 53.5 | [51.1,55.9] | 60.4 | [57.8,62.9] |  |  |  |  | 84.7 | [82.5,86.6] | 73.1 | [70.8, 75.3] |  |  |  |
| Bangladesh 2014 | 67.0 | [62.5,71.1] | 65.9 | [62.3,69.3] | 24.7 | [22.0,27.7] | 11.8 | [10.0, 13.8] | 86.6 | [83.5,89.1] | 70.9 | [68.3, 73.4] | 62.3 | [59.3,65.1] |  |
| India 2005-06 | 45.4 | [43.6,47.2] | n/a |  |  |  |  |  | 92.8 | [92.1,93.4] | n/a |  |  |  |  |
| India 2015-16 | 81.1 | [80.4,81.7] | n/a |  |  |  |  |  | 96.2 | [95.9,96.5] | n/a |  |  |  |  |
| Nepal 2006 | 47.5 | [42.6,52.5] | 19.2 | [15.2,23.9] |  |  |  |  | 78.5 | [73.7,82.7] | 73.3 | [69.7, 76.6] |  |  |  |
| Nepal 2011 | 65.5 | [61.2,69.5] | 34.8 | [30.9,38.8] |  |  |  |  | 82.5 | [78.4,86.1] | 55.6 | [50.7, 60.3] |  |  |  |
| Nepal 2016 | 82.5 | [79.0,85.6] | 56.7 | [52.7,60.7] | 56.7 | [52.7,60.7] | 24.4 | [20.5, 28.8] | 88.3 | [85.0,90.9] | 54.3 | [50.9, 57.6] | 47.1 | [43.7,50.5] |  |

Table S2. Percent of babies who received newborn care interventions among home births

Table S3. Antiseptic placed on the umbilical cord among home births

|  | **Chlorhexidine** | | **Antibiotic** | | **Antiseptic** | | **Spirit** | | **Gentian Violet** | |
| --- | --- | --- | --- | --- | --- | --- | --- | --- | --- | --- |
| **Survey** | **%** | **95% CI** | **%** | **95% CI** | **%** | **95% CI** | **%** | **95% CI** | **%** | **95% CI** |
| Bangladesh 2007 | na |  | 8.4 | [7.0,10.0] | 7.6 | [6.2,9.3] | 0.5 | [0.3,0.8] | 1.0 | [0.6,1.5] |
| Bangladesh 2011 | na |  | 10.6 | [9.4,12.0] | 8.4 | [7.2,9.7] | 0.4 | [0.2,0.7] | 0.5 | [0.2,0.9] |
| Bangladesh 2014 | 0.0 |  | 16.7 | [14.5,19.1] | 9.0 | [7.4,11.0] | 0.3 | [0.1,0.8] | 1.2 | [0.7,1.9] |
| Nepal 2011 | 0.8 | [0.4,1.6] | na |  | na |  |  |  | na |  |
| Nepal 2016 | 13.4 | [11.2,15.9] | na |  | na |  | 1.1 | [0.5,2.4] | na |  |
